# Arginine vasopressin deficiency following pembrolizumab-induced hypophysitis: a case report and review of the literature

**DOI:** 10.3389/fendo.2026.1976186

**Published:** 2026-10-23

**Authors:** Annina Rebecca Dold, Raphael Jeker, Michael Mark

**Affiliations:** 1 Department of Internal Medicine, Cantonal Hospital Grisons, Chur, Switzerland; 2 Department of Oncology and Hematology, Cantonal Hospital Grisons, Chur, Switzerland; 3 Faculty of Biomedical Sciences, Università della Svizzera Italiana, Lugano, Switzerland

**Keywords:** arginine vasopressin deficiency, case report, hypophysitis, immune checkpoint inhibitor, immune-related adverse events, pembrolizumab

## Abstract

Immune checkpoint inhibitors (ICIs) have revolutionized oncologic therapy by substantially improving survival outcomes in various malignancies. These agents—such as ipilimumab, nivolumab, pembrolizumab, atezolizumab, and durvalumab—modulate the immune system by inhibiting key checkpoint receptors, including cytotoxic T-lymphocyte antigen 4 (CTLA-4), programmed cell death protein 1 (PD-1), and programmed cell death ligand 1 (PDL-1), thereby facilitating tumor regression. However, immune modulation frequently triggers immune-related adverse events, which can affect any organ system, particularly the skin, liver, gastrointestinal tract, lung, and endocrine glands. Among the most common endocrine disorders induced by ICIs are thyroid, pituitary, adrenal, and beta-cell dysfunctions. Pituitary dysfunction is most commonly associated with combined CTLA-4 and PD-1/PDL-1 inhibitor regimens. This case report discusses a rare instance of presumed arginine vasopressin deficiency (AVP-D) secondary to pembrolizumab-monotherapy-induced hypophysitis, which was unmasked following glucocorticoid therapy. A 59-year-old male with metastatic lung adenocarcinoma treated with pembrolizumab developed gastrointestinal symptoms and was subsequently diagnosed with toxic colitis. Following the initiation of glucocorticoid therapy, he developed hypernatremia, polyuria, and polydipsia. Further evaluation suggested AVP-D, with rapid symptom resolution under desmopressin. This report underscores the complexity of endocrine disturbances associated with ICIs and the diagnostic challenges associated with glucocorticoid therapy in patients with ICI-related hypophysitis. The occurrence of arginine vasopressin deficiency in the context of pembrolizumab-induced hypophysitis is rare and not well investigated, highlighting the need for further research into the underlying mechanisms and optimal clinical management of these immune-mediated endocrine disorders.

## 1 Introduction

Immune checkpoint inhibitors (ICIs) represent a substantial advancement in oncologic therapy (1) and have led to improved survival outcomes in many malignancies (2).

ICIs modulate the immune system to enhance tumor regression through the inhibition of cytotoxic T lymphocyte antigen 4 (CTLA-4) (such as ipilimumab), programmed cell death protein 1 (PD1) (for example, nivolumab and pembrolizumab), and its ligand programmed cell death ligand 1 (PDL1) (e.g., atezolizumab and durvalumab) (3, 4). PD1 is a receptor specific to immune cells, and its ligands are proteins found on antigen-expressing cells and tumor cells. PD1 lowers the threshold for apoptosis and is involved in a signaling pathway that leads to T-cell destruction (5). Due to immune modulation, ICIs frequently trigger an exaggerated immune response, resulting in immune-related adverse events (2, 3). These side effects can affect any organ system (6), most commonly involving the skin, gastrointestinal, and endocrine systems (2). Among the most common endocrine disorders induced by ICIs (in descending order) are thyroid, pituitary, adrenal, and beta-cell dysfunctions (7). Hypophysitis is most commonly associated with combined CTLA-4 and PD-1/PDL-1 inhibitor regimens (5, 7, 8), with men more frequently affected by immunotherapy-induced hypophysitis (9, 10). Secondary adrenal insufficiency is the most frequent pathology resulting from ICI-induced hypophysitis, while arginine vasopressin deficiency remains rare (9, 11). The present case provides insight into a rare presentation of arginine vasopressin deficiency secondary to pembrolizumab monotherapy-induced hypophysitis, unmasked by glucocorticoid administration. Additionally, we systematically searched PubMed for case reports of hypophysitis following pembrolizumab monotherapy.

## 2 Literature search and case selection

A structured literature search was conducted to identify published case reports describing endocrine adverse events associated with pembrolizumab therapy, with particular emphasis on pituitary dysfunction and immune checkpoint inhibitor-induced hypophysitis. The PubMed/MEDLINE database was searched repeatedly between April 2025 and April 2026. Searches were restricted to articles published within the preceding 10 years and classified as case reports in PubMed.

The following search terms were applied individually: *“PD1 inhibitor-associated adverse events,” “PD1-inhibitor endocrine adverse events,” “pembrolizumab endocrine adverse events,” “immune-therapy related adverse events,” “pembrolizumab pituitary dysfunction,” pembrolizumab pituitary central diabetes insipidus,”and “pembrolizumab hypophysitis.”*

Titles and abstracts were screened for potentially relevant publications, followed by full-text assessment where appropriate. Case reports were considered eligible for further analysis if they described pembrolizumab administered as monotherapy and documented at least one impairment of a pituitary hormone axis. Reports involving pembrolizumab in combination with other immune checkpoint inhibitors or additional immunotherapeutic agents were excluded. Similarly, cases without evidence of pituitary hormonal dysfunction were excluded from the analysis.

Following application of the predefined eligibility criteria, a total of 32 case reports were included. Data from the eligible reports were subsequently reviewed with a focus on the affected pituitary hormone axes and the clinical and endocrine characteristics of pembrolizumab-associated pituitary dysfunction.

## 3 Case description

In 2024, a male patient in his 50s presented to the emergency department with a two-week history of diarrhea and vomiting, resulting in a weight loss of approximately 5 kg. He was diagnosed with metastatic pulmonary adenocarcinoma and had been receiving pembrolizumab (200 mg intravenously) for 12 months, completing a total of 19 cycles. His most recent administration was six days prior to hospital admission.

Initial diagnostic workup included stool cultures and testing for norovirus and *Clostridium difficile*, all of which tested negative. A computed tomography (CT) scan of the abdomen, performed 9 days before admission, showed no signs of gastrointestinal pathology. However, a subsequent colonoscopy revealed erythematous changes of the colonic wall, and biopsies of the terminal ileum and colon confirmed drug-induced toxic colitis (**Figure 1**). A follow-up CT scan of the abdomen showed diffuse wall thickening of the rectum and sigmoid colon, as well as a markedly distended stomach.

**Figure 1:**
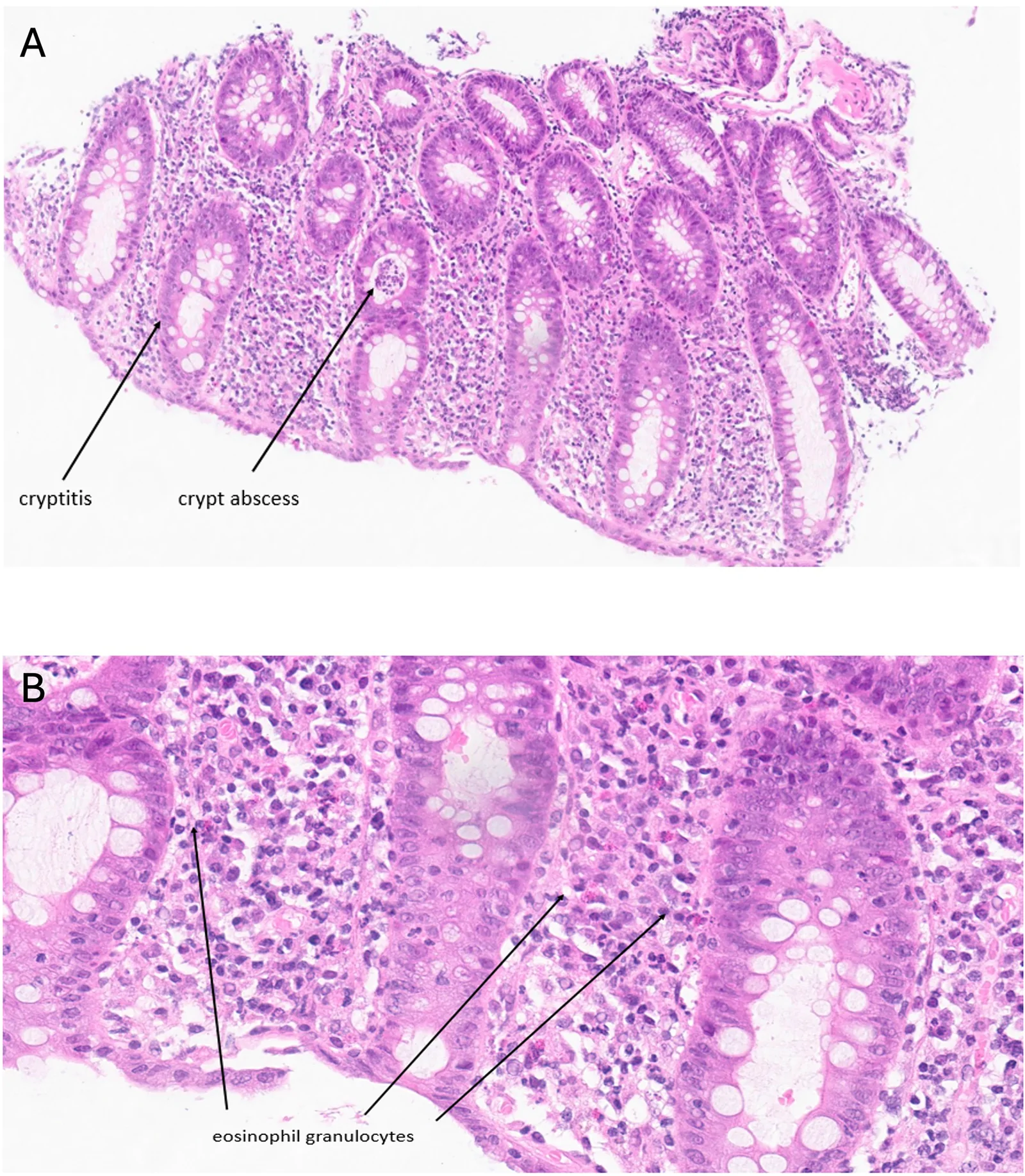
Histological findings of pembrolizumab-induced colitis. **(A)** Colonic mucosa showing cryptitis and crypt abscess formation. **(B)** Increased infiltration of the lamina propria by inflammatory cells, including eosinophilic granulocytes (arrows).

Due to concurrent gastroparesis with persistent vomiting, a triluminal tube was inserted 11 days after admission to provide gastric decompression and allow for enteral nutrition. Treatment was initiated with high-dose intravenous methylprednisolone (80 mg daily for 6 days), later transitioning to oral prednisolone (50 mg daily for 2 days, tapering to 30 mg daily for 4 days, and then 20 mg daily).

During his hospital stay, the patient exhibited substantial thirst, which was initially attributed to diarrhea and vomiting. The progressive development of polyuria was interpreted as secondary to increased fluid intake and substitution therapy, occurring simultaneously with a decrease in diarrhea following steroid therapy and reduced vomiting after insertion of the triluminal tube. Nine days after admission, the patient developed acute hypernatremia (161 mmol/L), occurring 3 days after initiation of glucocorticoid therapy. Initial treatment involved intravenous 5% glucose solution and free-water administration through the triluminal tube. However, these measures did not sufficiently reduce sodium levels. Laboratory findings showed serum hyperosmolarity (315 mOsm/kg) with concurrent hypo-osmolar urine (79 mmol/l). Arginine vasopressin deficiency was therefore suspected, prompting a trial of intranasal desmopressin (10 µg daily). This rapidly corrected the hypernatremia and polyuria, almost normalizing serum osmolality (298 mmol/kg) and increasing urine osmolality (132 mmol/l to 512 mmol/l in 1 day) (**Table 1**).

**Table 1:** Serial laboratory values of serum and urine electrolytes/osmolality during hospitalization.

Day after admission	Serum Sodium (136–146 mmol/l)	Serum Osmolality (280–300 mmol/kg)	Urine Sodium (50–150 mmol/l)	Urine Osmolality (770–1290 mmol/l)
Day 8	145	–	–	–
Day 9	**161**	**315**	–	**79**
Day 10	**159**	–	**< 20**	–
Day 11	**151**	–	**< 20**	**132**
Day 12	144	298	53	**512**

Values in bold red indicate pathologically abnormal levels relative to the reference range.

Further investigation revealed undetectable copeptin levels; however, the measurement was performed while serum sodium levels were within the normal range. Thyroid function tests indicated low-normal thyroid-stimulating hormone with free triiodothyronine and thyroxine levels below normal, suggesting possible central hypothyroidism, for which levothyroxine 0.1 mg was initiated. Additional hormonal testing revealed low follicle-stimulating hormone with normal luteinizing hormone and testosterone levels; therefore, no gonadal hormone substitution was initiated. Although adrenocorticotropic hormone (ACTH) levels were low, secondary adrenal insufficiency could not be definitively confirmed due to glucocorticoid-induced suppression of ACTH (**Table 2**) and the same applies for the diagnosis of central hypothyroidism, given the suppression of TSH under glucocorticoid therapy (12).

**Table 2:** Endocrine testing results at baseline presentation.

Parameter	Patient result	Reference range	Unit
fT4	**7.38**	13.1 – 21.3	pmol/l
fT3	**2.10**	4.09 – 6.65	pmol/l
TSH	0.46	0.30 – 3.18	mU/l
LH	3.10	1.7 – 8.6	IU/l
FSH	**2.60**	4.6 – 12.4	mU/ml
Testosterone	8.20	6.68 – 25.7	nmol/l
ACTH	**0.70**	1.3 – 13.9	pmol/l
Copeptin	2.70	< 13.8	pmol/l

Values in bold red indicate pathologically decreased levels below the reference range. fT4, free thyroxine; fT3, free triiodothyronine; TSH, thyroid-stimulating hormone; LH, luteinizing hormone; FSH, follicle-stimulating hormone; ACTH, adrenocorticotropic hormone.

Brain magnetic resonance imaging (MRI) conducted shortly after admission showed a normal sella turcica and pituitary gland, with no evidence of cerebral metastasis or pituitary enlargement. However, imaging was performed to exclude cerebral metastasis and was not performed especially to evaluate pituitary pathologies (**Figure 2**).

**Figure 2:**
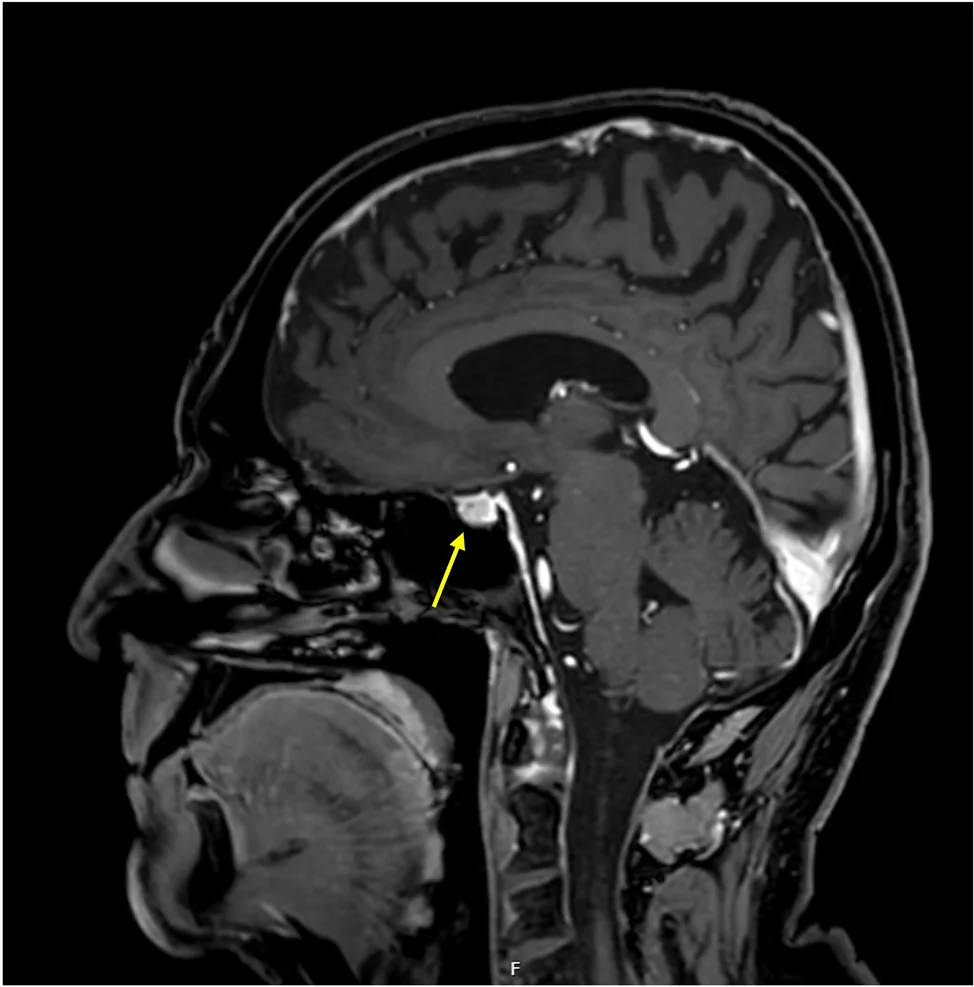
T1-weighted mDixon W KM image of the neurocranium with a normal pituitary gland (indicated by the yellow arrow).

During follow-up, the patient experienced three relapses of immune-mediated colitis during corticosteroid therapy. One relapse was complicated by hypovolemic shock and acute kidney injury, necessitating admission to the intensive care unit.

Treatment of the second relapse with infliximab led to an immediate allergic reaction, prompting discontinuation. Vedolizumab was subsequently administered during the third relapse with good tolerance.

In parallel, endocrine evaluation was performed to assess the patient’s endocrinopathy. At follow-up, the patient remained clinically stable under continued hormone replacement therapy with levothyroxine and desmopressin.

## 4 Discussion

ICI-induced hypophysitis is a well-recognized disorder associated with ICIs (5, 8). ICI-induced hypophysitis is most commonly observed with combined CTLA-4 and PD-1 inhibitor therapy (5, 7, 8), followed by CTLA-4 inhibitor monotherapy, and occurs least frequently with PD-1/PD-L1 inhibitor monotherapy (2, 11). Secondary adrenal insufficiency is the most frequent pathology resulting from ICI-induced hypophysitis, while arginine vasopressin deficiency remains rare (9, 11). Limited data are available regarding the incidence of hypophysitis associated with pembrolizumab monotherapy, particularly in cases presenting with central diabetes insipidus. Given the limited data on the incidence of hypophysitis associated with pembrolizumab monotherapy, we conducted a targeted literature search specifically focusing on pembrolizumab-associated hypophysitis.

Our review of published case reports (see **Supplementary Material**) indicated that the PD-1 inhibitor pembrolizumab, when triggering hypophysitis, often affects multiple hormonal axes, with the adrenal axis being the most frequently impacted (13–42), whereas case reports of arginine vasopressin deficiency following pembrolizumab therapy stayed rare (43, 44). **Table 3** summarizes all comparable cases identified in our literature search that describe arginine vasopressin deficiency associated with pembrolizumab monotherapy. We identified five case reports describing secondary hypothyroidism associated with pembrolizumab-induced hypophysitis (15, 17, 21, 29, 30). Although secondary hypothyroidism is not uncommon during PD-1 inhibitor therapy (45), PD-1 inhibitors are more frequently associated with primary hypothyroidism (46).

**Table 3:** Previously published case reports with pembrolizumab-induced arginine vasopressin deficiency.

Author & year	Study title	Affected hormonal axes	MRI findings	Presenting symptoms
Alasmar et al. (2026) (44)	Central Diabetes Insipidus Following Steroid Replacement for Hypoadrenalism in Pembrolizumab-Induced Hypophysitis	Arginine vasopressin deficiency, secondary adrenal insufficiency	T1-weighted MRI with homogeneous enhancement and expansion of the pituitary gland and stalk	Polyuria
Yong et al. (2023) (43)	Pembrolizumab-related central diabetes insipidus	Arginine vasopressin deficiency	Neuroimaging weighted images without an infiltrative lesion	Dyspnea, preceded by a 2-week history of malaise

Patients receiving PD-1 inhibitor monotherapy more frequently experience symptoms such as fatigue, loss of appetite, and myalgia, whereas patients treated with the CTLA-4 inhibitor ipilimumab are more likely to present with headache (47). These findings are concordant with the symptoms described in the reviewed case reports, which mostly report nonspecific symptoms such as anorexia and/or fatigue before the diagnosis of hypopituitarism (13–26, 28–30, 33–35, 39–42, 48). Similarly, our patient exhibited marked fatigue and loss of appetite before the diagnosis of hypophysitis, although these symptoms could also be attributed to the underlying malignancy and immune-mediated colitis.

MRI is the standard modality for investigating structural pituitary pathology. In the majority of patients, ICI-induced hypophysitis is associated with pituitary enlargement and hypoenhancing lesions of the anterior pituitary lobe (49). However, MRI scans performed within 1 month of diagnosis have shown substantially less pituitary swelling in patients treated with PD-1 inhibitors than in those treated with the CTLA-4 inhibitor ipilimumab (47). Consistent with this, MRI findings in our case and most case reports in our review did not describe pituitary abnormalities on MRI (13, 14, 18, 19, 22–25, 30, 34, 36–39, 41, 42, 48). The absence of typical radiological findings may contribute to delayed or missed diagnosis. As a limiting factor should be noted that the MRI in our case was performed shortly before the diagnosis of arginine vasopressin deficiency and was not specifically acquired using a dedicated high-resolution sella MRI with thin cuts. This limitation substantially reduces the diagnostic value of our MRI findings, as subtle lesions of the posterior pituitary stalk or loss of the normal T1-hyperintense bright spot may be missed using a standard brain MRI protocol.

In our patient, arginine vasopressin deficiency was suspected only after initiation of glucocorticoid therapy. Several case reports reviewed by Yang et al. have similarly described the onset of arginine vasopressin deficiency following glucocorticoid administration (50). Evidence suggests that the antidiuretic effect of anti-diuretic hormone (ADH) in arginine vasopressin deficiency may be compensated by concomitant cortisol deficiency through both ADH-dependent and ADH-independent mechanisms (50–53).

First, cortisol appears to reduce the sensitivity of renal V2 receptors to ADH, thereby decreasing the number of aquaporin channels inserted into collecting duct cells (51). Consequently, cortisol deficiency enhances ADH activity (50). Second, corticotropin-releasing hormone (CRH) stimulates both ACTH and ADH secretion. In the absence of cortisol, CRH can increase ADH release through negative feedback mechanisms (50). Third, cortisol exerts mineralocorticoid-like effects that promote renal sodium reabsorption. Cortisol deficiency leads to sodium and volume depletion, indirectly stimulating ADH secretion. Lastly, cortisol has vasoconstrictive effects that increase preload and stroke volume; when cortisol is deficient, activation of the renin-angiotensin-aldosterone system may further stimulate ADH release (51).

In addition, cortisol influences renal function by modulating glomerular filtration rate (GFR) and tubular water permeability. Cortisol deficiency reduces GFR and decreases renal sodium and water excretion, further stimulating ADH secretion (51).

Although secondary adrenal insufficiency could not be definitively confirmed biochemically in our patient, as ACTH levels were measured only 7 days after initiation of glucocorticoid therapy, precluding a clear distinction between secondary adrenal insufficiency and purely iatrogenic adrenal insufficiency, the temporal association between glucocorticoid initiation and development of hypernatremia and polyuria after three days of therapy is compatible with glucocorticoid-induced unmasking of arginine vasopressin deficiency in the setting of possible pre-existing hypocortisolism. Nevertheless, arginine vasopressin deficiency can’t be substantiated solely by the clinical course and reduced copeptin level measurement, as the measurement was conducted under normal sodium levels, and no hypertonic saline infusion test was performed. It should be acknowledged that colitis, with substantial concurrent fluid losses due to diarrhea and vomiting, initially provided an alternative explanation for the patient’s thirst and hypernatremia and thereby delayed evaluation for arginine vasopressin deficiency. Progressive polyuria was initially interpreted as secondary to increased fluid intake and substitution therapy. It also remains possible that the substantial fluid losses associated with colitis contributed to the development of hypernatremia.

Despite these limitations, our patient maintained normal serum sodium levels, after hospitalization and after complete resolution of the colitis, while on desmopressin therapy, providing support of our hypothesis of arginine vasopressin deficiency.

In our case, no assessment of the pituitary hormone axes, apart from TSH measurement, was performed prior to initiation of immunotherapy, which further complicated the diagnosis of secondary hypocortisolism. As described above, hypophysitis is a well-established complication of immune checkpoint inhibitor therapy and is associated with nonspecific symptoms (5, 8). In the absence of baseline hormone levels, the detection of subsequent hormonal abnormalities is considerably more challenging. Baseline endocrine assessment may therefore facilitate interpretation of subsequent hormonal abnormalities. However, routine comprehensive pituitary screening in all asymptomatic patients receiving PD-1 inhibitor monotherapy is not uniformly recommended, and more extensive pituitary assessment should particularly be considered in high-risk patients and in patients presenting with suggestive symptoms (54).

## 5 Conclusion

- Secondary adrenal insufficiency may obscure the clinical presentation of arginine vasopressin deficiency by enhancing ADH activity via several mechanisms, including increased ADH sensitivity, reduced aquaporin-2 expression in renal tubules, and volume depletion-mediated stimulation of ADH release. Consequently, arginine vasopressin deficiency may only manifest following glucocorticoid replacement therapy.
- Patients receiving PD-1 inhibitors rarely exhibit structural pituitary abnormalities on MRI. The absence of typical radiological findings may contribute to delayed or missed diagnosis.
- Immune checkpoint inhibitors can trigger diverse immune-mediated inflammatory processes, which, when occurring concurrently, may contribute to a delayed or challenging diagnosis, highlighting the need for low threshold evaluation of pituitary hormone deficiencies in patients presenting with nonspecific symptoms.
- The occurrence of arginine vasopressin deficiency in the context of pembrolizumab-induced hypophysitis is rare and not well investigated, highlighting the need for further research into the underlying mechanisms and optimal clinical management of these immune-mediated endocrine disorders.

## 6 Patient perspective

At the onset, my symptoms manifested as an excessive urge to drink fluids (approximately 7–8 liters per day) and severe diarrhea. Unfortunately, I was no longer able to control either condition, which was highly distressing and concerning for me.

When I was subsequently admitted to the hospital, it marked a very difficult period characterized by many uncertainties. Due to the complexity of my condition, the physicians had to explore numerous approaches in order to identify an appropriate treatment strategy. Throughout this process, I was consistently well informed and thoroughly educated by the treating medical team regarding each subsequent step.

As a result, I was able to endure the prolonged period of 11 weeks in the general ward, intermediate care unit (IMC), and intensive care unit (ICU) without fear, as I felt that I was in very capable hands. Even during times when uncertainty among the medical staff was perceptible, this was always communicated transparently, which reassured me and strengthened my confidence that the physicians would not give up on my case.

When a medication was eventually proposed on a trial basis as a potential solution for managing the side effects of the administered immunotherapy, this brought me great relief and a strong sense of gratitude toward the entire medical team. Despite the uncertainty regarding the likelihood of success of this therapeutic approach, I remained optimistic and was willing to pursue this option.

From the patient’s perspective, it was particularly important and reassuring that the interdisciplinary collaboration between the responsible chief physician, attending physicians, and the various specialties, such as oncology, always functioned effectively.

## Data Availability

The original contributions presented in the study are included in the article/**Supplementary Material**, further inquiries can be directed to the corresponding author/s.

## References

[1] Zhao C Tella SH Del Rivero J Kommalapati A Ebenuwa I Gulley J . Anti-PD-L1 treatment induced central diabetes insipidus. J Clin Endocrinol Metab. (2018) 103:365–9. doi: 10.1210/jc.2017-01905 PMC5800826 29220526

[2] Barnabei A Strigari L Corsello A Paragliola RM Falzone L Salvatori R . Immune checkpoint inhibitor-induced central diabetes insipidus: Looking for the needle in the haystack or a very rare side-effect to promptly diagnose? Front Oncol. (2022) 12:798517. doi: 10.3389/fonc.2022.798517 35311088 PMC8927719

[3] Levy M Abeillon J Dalle S Assaad S Borson-Chazot F Disse E . Anti-PD1 and anti-PDL1-induced hypophysitis: A cohort study of 17 patients with longitudinal follow-up. J Clin Med. (2020) 9. doi: 10.3390/jcm9103280 33066179 PMC7601962

[4] Bai X Chen X Wu X Huang Y Zhuang Y Chen Y . Immune checkpoint inhibitor-associated pituitary adverse events: An observational, retrospective, disproportionality study. J Endocrinol Invest. (2020) 43:1473–83. doi: 10.1007/s40618-020-01226-4 32239475

[5] Byun DJ Wolchok JD Rosenberg LM Girotra M . Cancer immunotherapy - immune checkpoint blockade and associated endocrinopathies. Nat Rev Endocrinol. (2017) 13:195–207. doi: 10.1038/nrendo.2016.205 28106152 PMC5629093

[6] Ramos-Casals M Brahmer JR Callahan MK Flores-Chavez A Keegan N Khamashta MA . Immune-related adverse events of checkpoint inhibitors. Nat Rev Dis Primers. (2020) 6:38. doi: 10.1038/s41572-020-0160-6 32382051 PMC9728094

[7] de Filette J Andreescu CE Cools F Bravenboer B Velkeniers B . A systematic review and meta-analysis of endocrine-related adverse events associated with immune checkpoint inhibitors. Horm Metab Res. (2019) 51:145–56. doi: 10.1055/a-0843-3366 30861560

[8] Wang DY Salem JE Cohen JV Chandra S Menzer C Ye F . Fatal toxic effects associated with immune checkpoint inhibitors: A systematic review and meta-analysis. JAMA Oncol. (2018) 4:1721–8. doi: 10.1001/jamaoncol.2018.3923 30242316 PMC6440712

[9] Amereller F Deutschbein T Joshi M Schopohl J Schilbach K Detomas M . Differences between immunotherapy-induced and primary hypophysitis-a multicenter retrospective study. Pituitary. (2022) 25:152–8. doi: 10.1007/s11102-021-01182-z PMC8821509 34518996

[10] Mahzari M Liu D Arnaout A Lochnan H . Immune checkpoint inhibitor therapy associated hypophysitis. Clin Med Insights Endocrinol Diabetes. (2015) 8:21–8. doi: 10.4137/cmed.s22469 25861234 PMC4376202

[11] Barroso-Sousa R Barry WT Garrido-Castro AC Hodi FS Min L Krop IE . Incidence of endocrine dysfunction following the use of different immune checkpoint inhibitor regimens: A systematic review and meta-analysis. JAMA Oncol. (2018) 4:173–82. doi: 10.1001/jamaoncol.2017.3064 28973656 PMC5838579

[12] Rubello D Sonino N Casara D Girelli ME Busnardo B Boscaro M . Acute and chronic effects of high glucocorticoid levels on hypothalamic-pituitary-thyroid axis in man. J Endocrinol Invest. (1992) 15:437–41. doi: 10.1007/bf03348767 1328349

[13] Alfares K Han HJ . Pembrolizumab-induced isolated adrenocorticotropic hormone (ACTH) deficiency. Cureus. (2024) 16:e52235. doi: 10.7759/cureus.52235 38352096 PMC10861847

[14] Baldawi MB Dhami B Gosai J Al-Khafaji RH . Adverse endocrine-related effects of pembrolizumab precipitating severe hyponatremia. Cureus. (2022) 14:e28393. doi: 10.7759/cureus.28393 36171843 PMC9508894

[15] Balti E Verhaeghe S Kruse V Roels S Coremans P . Exploring a new entity of single-agent pembrolizumab-associated hypophysitis. Cureus. (2022) 14:e27763. doi: 10.7759/cureus.27763 36127991 PMC9481187

[16] Cheng NH Lee H Balchander D Mimms R Krishnamurthy M . Immunotherapy induced adrenal insufficiency: An underdiagnosed cause of persistent hypotension in cancer. J Community Hosp Intern Med Perspect. (2024) 14:68–70. doi: 10.55729/2000-9666.1375 PMC11464065 39391114

[17] Chowdhury RH Hasan MN . Pembrolizumab induced hypophysitis in a patient allergic to triamcinolone: An increasingly recognized endocrinopathy related to immunotherapy. Mymensingh Med J. (2020) 29:1021–5. 33116112

[18] Doodnauth AV Klar M Mulatu YS Malik ZR Patel KH McFarlane SI . Pembrolizumab-induced hypophysitis with isolated adrenocorticotropic hormone (ACTH) deficiency: A rare immune-mediated adverse event. Cureus. (2021) 13:e15465. doi: 10.7759/cureus.15465 34123679 PMC8186842

[19] El Alam A Fleifel M El Masri D Nassani BM Abou Chaaya J Minkailou M . A case of myxedema coma and adrenal insufficiency post pembrolizumab. Case Rep Endocrinol. (2024) 2024:5444975. doi: 10.1155/2024/5444975 39021640 PMC11254456

[20] Escano L Desai P . Secondary adrenal insufficiency as an immune-related adverse event of pembrolizumab therapy. Cureus. (2024) 16:e65163. doi: 10.7759/cureus.65163 PMC11339576 39176308

[21] Fernando A Mittal A Cheema R . Nothing great comes without its risks: A rare case of pembrolizumab-induced hypophysitis. JCEM Case Rep. (2024) 2:luad135. doi: 10.1210/jcemcr/luad135 PMC10729852 38116159

[22] Fujimiya T Azuma K Togashi Y Kuwata K Unezaki S Takeuchi H . Pembrolizumab-induced secondary adrenal insufficiency due to adrenocorticotrophic hormone deficiency in a patient with non-small-cell lung carcinoma: A case report. J Pharm Health Care Sci. (2024) 10:10. doi: 10.1186/s40780-024-00332-2 38365819 PMC10873928

[23] Hashinokuchi A Haro A Minagawa R Haruta Y Kawano H Suehiro T . Hypothyroidism with ACTH deficiency during pembrolizumab therapy for lung cancer: Case report and literature review. Cancer Diagn Progn. (2023) 3:498–503. doi: 10.21873/cdp.10246 37405219 PMC10316054

[24] Hinata Y Ohara N Sakurai Y Koda R Yoneoka Y Takada T . Isolated adrenocorticotropic hormone deficiency associated with severe hyperkalemia during pembrolizumab therapy in a patient with ureteral cancer and an ileal conduit: A case report and literature review. Am J Case Rep. (2021) 22:e931639. doi: 10.12659/ajcr.931639 34262010 PMC8297058

[25] Iijima H Sakai A Ebisumoto K Yamauchi M Teramura T Yamazaki A . Metachronous immune-related adverse events involving type 1 diabetes and isolated adrenocorticotropic hormone deficiency associated with pembrolizumab monotherapy for metastatic head and neck cancer: A case report. J Med Case Rep. (2023) 17:387. doi: 10.1186/s13256-023-04106-6 37697390 PMC10496339

[26] Inoue S Tsunoda Y Yamamoto K Okamoto H . Immunoglobulin G4-related autoimmune pancreatitis and hypopituitarism following immune checkpoint inhibitor therapy: A case report. Intern Med. (2024) 64:209–15. doi: 10.2169/internalmedicine.3591-24 PMC11802208 38811225

[27] Iwamoto Y Tatsumi F Ohnishi M Katakura Y Kimura T Shimoda M . A case of empty sella syndrome with adrenal insufficiency masked by prednisolone after administration of immune checkpoint inhibitors. Med (Baltimore). (2024) 103:e37204. doi: 10.1097/md.0000000000037204 38457550 PMC10919508

[28] Kafley S Tamrakar S Samra M Shanmugar S Gupta I . Immune checkpoint inhibitors and endocrine disruption: A case of hyponatremia and adrenal insufficiency. Cureus. (2024) 16:e70089. doi: 10.7759/cureus.70089 39449870 PMC11500490

[29] Khan WJ Asif M Chaudhry HS Aslam S Nadeem I . Pembrolizumab-induced broken heart syndrome, pneumonitis, and hypophysitis occurring concurrently; A deadly triad. J Community Hosp Intern Med Perspect. (2023) 13:88–90. doi: 10.55729/2000-9666.1173 37877053 PMC10593175

[30] Khatri S Alom M Kashfi S Atallah J Goswami G . Simultaneous presentation of secondary adrenal insufficiency and primary hypothyroidism due to pembrolizumab: A case report. J Investig Med High Impact Case Rep. (2023) 11:23247096231194401. doi: 10.1177/23247096231194401 37642143 PMC10467221

[31] Montero Perez O Sanchez Escudero L Guzman Ramos MI Avino Tarazona V . Hypophysitis secondary to pembrolizumab: A case report and review of the literature. Anticancer Drugs. (2022) 33:94–9. doi: 10.1097/CAD.0000000000001129 34261922

[32] Moreira I Vilarica AS Alves P . First report of a very rare complication in a case of nonsmall cell lung carcinoma. Breathe (Sheff). (2021) 17:210107. doi: 10.1183/20734735.0107-2021 35035567 PMC8753643

[33] Nakagami C Endoh M Nakatsuka M Nakahashi K Ota H Aso M . Pituitary hypoadrenocorticism and hypothyroidism after immunochemotherapy followed by salvage surgery for lung cancer: A case report. Gen Thorac Cardiovasc Surg cases. (2023) 2:7. doi: 10.1186/s44215-022-00019-w 39516983 PMC11533550

[34] Nakamura T Imai R Nishimura N . A case of nonsmall-cell lung cancer with anaphylaxis after 41 courses of pembrolizumab along with adrenal insufficiency as an immune-related adverse event. Case Rep Oncol. (2022) 15:804–8. doi: 10.1159/000526561 36825102 PMC9941790

[35] Oguz SH Unluturk U Aksoy S Erbas T . Clinical course and management of pembrolizumab-associated isolated adrenocorticotrophic hormone deficiency: A new case and literature review. Immunotherapy. (2021) 13:1157–63. doi: 10.2217/imt-2021-0061 34387129

[36] Oristrell G Baneras J Ros J Munoz E . Cardiac tamponade and adrenal insufficiency due to pembrolizumab: A case report. Eur Heart J Case Rep. (2018) 2:yty038. doi: 10.1093/ehjcr/yty038 31020118 PMC6177032

[37] Porntharukchareon T Tontivuthikul B Sintawichai N Srichomkwun P . Pembrolizumab- and ipilimumab-induced diabetic ketoacidosis and isolated adrenocorticotropic hormone deficiency: A case report. J Med Case Rep. (2020) 14:171. doi: 10.1186/s13256-020-02502-w 32988414 PMC7523376

[38] Rossi S Silvetti F Bordoni M Ciarloni A Salvio G Balercia G . Pembrolizumab-induced thyroiditis, hypophysitis and adrenalitis: A case of triple endocrine dysfunction. JCEM Case Rep. (2024) 2:luae200. doi: 10.1210/jcemcr/luae200 39498471 PMC11532647

[39] Tanabe J Watanabe N Ito M Kanasaki K Tanabe K . Differentiating immune-related adrenal insufficiency from low cardiac output syndrome: A case report. Cureus. (2022) 14:e31349. doi: 10.7759/cureus.31349 36523682 PMC9744394

[40] Tanaka Y Amano T Takahashi A Nishimura H Yamanaka H Yoneoka Y . Locally advanced endometrial cancer with multiple immune-related adverse events coinciding with the complete response to radiotherapy after immune checkpoint inhibitor therapy: A case report. Gynecol Oncol Rep. (2023) 49:101265. doi: 10.1016/j.gore.2023.101265 37705723 PMC10495623

[41] Yamagata S Kageyama K Takayasu S Asari Y Makita K Terui K . Progression of hypopituitarism and hypothyroidism after treatment with pembrolizumab in a patient with adrenal metastasis from non-small-cell lung cancer. Intern Med. (2019) 58:3557–62. doi: 10.2169/internalmedicine.3008-19 31462588 PMC6949454

[42] Yamamoto K Tokumasu K Oka K Hasegawa K Otsuka F . Isolated adrenocorticotropin deficiency induced by pembrolizumab for hypopharyngeal cancer: A case report. Clin Case Rep. (2021) 9:e04305. doi: 10.1002/ccr3.4305 34136239 PMC8190675

[43] Chen Han Yong AA Parind V Christopher H . Pembrolizumab related central diabetes insipidus. J Clin Rev Case Rep. (2023) 8:64–6.

[44] Alasmar A San SS Ta T Mani SE . Central diabetes insipidus following steroid replacement for hypoadrenalism in pembrolizumab-induced hypophysitis. Cureus. (2026) 18:e107752. doi: 10.7759/cureus.107752 42205670 PMC13211477

[45] Wright JJ Powers AC Johnson DB . Endocrine toxicities of immune checkpoint inhibitors. Nat Rev Endocrinol. (2021) 17:389–99. doi: 10.1038/s41574-021-00484-3 33875857 PMC8769055

[46] Chang LS Barroso-Sousa R Tolaney SM Hodi FS Kaiser UB Min L . Endocrine toxicity of cancer immunotherapy targeting immune checkpoints. Endocr Rev. (2019) 40:17–65. doi: 10.1210/er.2018-00006 PMC6270990 30184160

[47] Faje A Reynolds K Zubiri L Lawrence D Cohen JV Sullivan RJ . Hypophysitis secondary to nivolumab and pembrolizumab is a clinical entity distinct from ipilimumab-associated hypophysitis. Eur J Endocrinol. (2019) 181:211–9. doi: 10.1530/eje-19-0238 31176301

[48] Sonehara K Tateishi K Hirabayashi T Araki T Ikuyama Y Machida R . A case of lung adenocarcinoma with long-term response after late-onset pembrolizumab-induced acute adrenal insufficiency. Case Rep Oncol. (2021) 14:1–7. doi: 10.1159/000508068 33613234 PMC7879310

[49] Kurokawa R Ota Y Gonoi W Hagiwara A Kurokawa M Mori H . MRI findings of immune checkpoint inhibitor-induced hypophysitis: Possible association with fibrosis. AJNR Am J Neuroradiol. (2020) 41:1683–9. doi: 10.3174/ajnr.a6692 32763900 PMC7583108

[50] Yang LY Lin S Xie QB Yin G . Central diabetes insipidus unveiled by glucocorticoid therapy in a patient with an empty sella: A case report and literature review. Med (Baltimore). (2020) 99:e22939. doi: 10.1097/md.0000000000022939 33120853 PMC7581106

[51] Castle-Kirszbaum M Goldschlager T Shi MDY Fuller PJ . Glucocorticoids and water balance: Implications for hyponatremia management and pituitary surgery. Neuroendocrinology. (2023) 113:785–94. doi: 10.1159/000530701 37062279 PMC10389798

[52] Green HH Harrington AR Valtin H . On the role of antidiuretic hormone in the inhibition of acute water diuresis in adrenal insufficiency and the effects of gluco- and mineralocorticoids in reversing the inhibition. J Clin Invest. (1970) 49:1724–36. doi: 10.1172/jci106390 5449709 PMC322656

[53] Linas SL Berl T Robertson GL Aisenbrey GA Schrier RW Anderson RJ . Role of vasopressin in the impaired water excretion of glucocorticoid deficiency. Kidney Int. (1980) 18:58–67. doi: 10.1038/ki.1980.110 7218660

[54] Yuen KCJ Samson SL Bancos I Gosmanov AR Jasim S Fecher LA . American Association of Clinical Endocrinology Disease State Clinical Review: Evaluation and management of immune checkpoint inhibitor-mediated endocrinopathies: A practical case-based clinical approach. Endocr Pract. (2022) 28:719–31. doi: 10.1016/j.eprac.2022.04.010 35477029

