# Supplementary material for: Arginine vasopressin deficiency following pembrolizumab-induced hypophysitis: a case report and review of the literature
Source: Front Endocrinol (Lausanne). 2026 Oct 23;17:1976186. doi: 10.3389/fendo.2026.1976186 (PMC13739506; doi:10.3389/fendo.2026.1976186)
Supplement: Supplementary file 1 [file Table1.docx]

**Literature Review: Pembrolizumab-Induced Hypophysitis & Endocrinopathies**

Tabelle 1: Konsolidierte Übersicht aller 32 veröffentlichten Fallberichte zu Pembrolizumab-assoziierter Hypophysitis, betroffenen hormonellen Achsen, MRT-Befunden und klinischer Symptomatik.

| **Author** | **Title** | **Affected hypophysitis-related hormonal axes** | **MRT report** | **Initial symptoms** |
| --- | --- | --- | --- | --- |
| **Alasmar et al. (2026) (1)** | Central Diabetes Insipidus Following Steroid Replacement for Hypoadrenalism in Pembrolizumab-Induced Hypophysitis | Central diabetes insipidus, secondary adrenal insufficiency | T1-weighted MRI with homogeneous enhancement and expansion of the pituitary gland and stalk | Polyuria |
| **Alfares & Han (2020) (2)** | Pembrolizumab-Induced Isolated Adrenocorticotropic Hormone (ACTH) Deficiency. | Secondary adrenal insufficiency | Normal pituitary gland on MRI | Poor oral intake, generalized fatigue, and difficulty ambulating |
| **Baldawi et al. (2021) (3)** | Adverse Endocrine-Related Effects of Pembrolizumab Precipitating Severe Hyponatremia. | Secondary adrenal insufficiency | Normal pituitary gland on MRI | Poor oral intake, nausea, vomiting, diarrhea |
| **Balti et al. (2020) (4)** | Exploring a New Entity of Single-Agent Pembrolizumab-Associated Hypophysitis. | Secondary adrenal insufficiency, secondary hypothyroidism, gonadotrophs hypogonadism | Enlarged pituitary gland with a homogeneous enhancement of the gland and pituitary stalk | Headache, nausea, and fatigue |
| **Cheng et al. (2019) (5)** | Immunotherapy-Induced Adrenal Insufficiency: An Underdiagnosed Cause of Persistent Hypotension in Cancer. | Secondary adrenal insufficiency | MRI contraindicated in the presence of tissue expanders used in breast reconstruction. | Generalized weakness |
| **Chowdhury & Hasan (2021) (6)** | Pembrolizumab-Induced Hypophysitis in a Patient Allergic to Triamcinolone: An Increasingly Recognized Endocrinopathy Related to Immunotherapy | Secondary adrenal insufficiency, secondary hypothyroidism | Keine Angaben im Abstract verfügbar | Unintentional loss of weight, easy fatigability, and breast pain while showering |
| **Doodnauth et al. (2021) (7)** | Pembrolizumab-Induced Hypophysitis With Isolated Adrenocorticotropic Hormone (ACTH) Deficiency | Secondary adrenal insufficiency | Normal pituitary gland on MRI | Worsening generalized fatigue, appetite loss, abdominal pain, and altered mental status |
| **El Alam et al. (2020) (8)** | A Case of Myxedema Coma and Adrenal Insufficiency Post Pembrolizumab. | Secondary adrenal insufficiency | Normal pituitary gland on MRI | Dysarthria, mild-to-moderate confusion, and psychomotor slowing for the past 4 h. Worsening fatigue and generalized edema for the past month |
| **Escaño & Desai (2021) (9)** | Secondary Adrenal Insufficiency as an Immune-Related Adverse Event of Pembrolizumab Therapy. | Secondary adrenal insufficiency | Keine Angaben verfügbar | Decreased appetite, nausea, and generalized weakness |
| **Fernando et al. (2020) (10)** | Nothing Great Comes Without Its Risks: A Rare Case of Pembrolizumab-Induced Hypophysitis. | Secondary adrenal insufficiency, hypogonadotropic hypogonadism, secondary hypothyroidism | Enlarged pituitary gland | Fatigue and cold intolerance, constipation, dry skin, weight gain, poor libido, erectile dysfunction, hot flashes, diaphoresis, and flushing |
| **Fujimiya et al. (2021) (11)** | Pembrolizumab-induced secondary adrenal insufficiency due to adrenocorticotropic hormone deficiency in a patient with non-small-cell lung carcinoma: a case report. | Secondary adrenal insufficiency | Normal pituitary gland on MRI | Fatigue, anorexia |
| **Hashinokuchi et al. (2021) (12)** | Hypothyroidism With ACTH Deficiency During Pembrolizumab Therapy for Lung Cancer: Case Report and Literature Review. | Secondary adrenal insufficiency | Normal pituitary gland on MRI | General fatigue |
| **Hinata et al. (2021) (13)** | Isolated Adrenocorticotropic Hormone Deficiency Associated with Severe Hyperkalemia During Pembrolizumab Therapy in a Patient with Ureteral Cancer and an Ileal Conduit: A Case Report and Literature Review. | Secondary adrenal insufficiency | Normal pituitary gland on MRI | Anorexia, general weakness, muscle pain |
| **Iijima et al. (2021) (14)** | Metachronous immune-related adverse events involving type 1 diabetes and isolated adrenocorticotropic hormone deficiency associated with pembrolizumab monotherapy for metastatic head and neck cancer: a case report. | Secondary adrenal insufficiency | Normal pituitary gland on MRI | Nausea and general fatigue for 1 week |
| **Inoue et al. (2021) (15)** | Immunoglobulin G4-Related Autoimmune Pancreatitis and Hypopituitarism Following Immune Checkpoint Inhibitor Therapy: A Case Report. | Secondary adrenal insufficiency | Magnetic resonance imaging (MRI) revealed a reduction in the signal intensity in the posterior pituitary lobe, but no signs of enlargement of the pituitary or pituitary stalk were identified. | Loss of appetite |
| **Iwamoto et al. (2020) (16)** | A case of Empty Sella syndrome with adrenal insufficiency masked by prednisolone after administration of immune checkpoint inhibitors | Secondary adrenal insufficiency | Empty sella syndrome | General malaise, edema of the lower extremities |
| **Kafley et al. (2021) (17)** | Immune Checkpoint Inhibitors and Endocrine Disruption: A Case of Hyponatremia and Adrenal Insufficiency. | Secondary adrenal insufficiency | Keine Angaben verfügbar | Non-projectile vomiting, generalized dull abdominal pain, several-week history of poor appetite and malaise. |
| **Khan et al. (2020) (18)** | Pembrolizumab-Induced Broken Heart Syndrome, Pneumonitis, and Hypophysitis Occurring Concurrently; A Deadly Triad. | Secondary adrenal insufficiency, secondary hypothyroidism, gonadotropic hypogonadism | Keine Angaben verfügbar | Shortness of breath and generalized weakness |
| **Khatri et al. (2021) (19)** | Simultaneous Presentation of Secondary Adrenal Insufficiency and Primary Hypothyroidism due to Pembrolizumab: A Case Report. | Secondary adrenal insufficiency, secondary hypothyroidism | Normal pituitary gland on MRI | Altered mental status and fatigue |
| **Montero et al. (2020) (20)** | Hypophysitis secondary to pembrolizumab: a case report and review of the literature. | Keine spezifischen Angaben im Abstract | Keine Angaben im Abstract verfügbar | Unspecific symptoms |
| **Moreira et al. (2020) (21)** | First report of a very rare complication in a case of non-small cell lung carcinoma. | Secondary adrenal insufficiency | Pituitary magnetic resonance imaging (MRI) showed diffuse substantial gadolinium enhancement, T2 hyperintensity | Asthenia, dizziness, mental confusion, and back pain with progressive worsening |
| **Nakagami et al. (2021) (22)** | Pituitary hypoadrenocorticism and hypothyroidism after immunochemotherapy followed by salvage surgery for lung cancer: a case report. | Secondary adrenal insufficiency | Enlarged pituitary gland | Anorexia |
| **Nakamura et al. (2021) (23)** | A Case of Non-small-Cell Lung Cancer with Anaphylaxis after 41 Courses of Pembrolizumab along with Adrenal Insufficiency as an Immune-Related Adverse Event. | Secondary adrenal insufficiency | Normal pituitary gland on MRI | Vomiting and anorexia |
| **Oğuz et al. (2021) (24)** | Clinical course and management of pembrolizumab-associated isolated adrenocorticotropic hormone deficiency: a new case and literature review. | Secondary adrenal insufficiency | Enlarged pituitary gland | Weakness and nausea/vomiting |
| **Oristrell et al. (2020) (25)** | Cardiac tamponade and adrenal insufficiency due to pembrolizumab: a case report. | Secondary adrenal insufficiency | Normal pituitary gland on MRI | Hypotension |
| **Rossi et al. (2021) (26)** | Pembrolizumab-induced Thyroiditis, Hypophysitis, and Adrenalitis: A Case of Triple Endocrine Dysfunction | Secondary adrenal insufficiency, hypogonadotropic hypogonadism | Normal pituitary gland on MRI | Fever, constipation, dizziness, asthenia, headache, and nausea |
| **Sonehara et al. (2020) (27)** | A Case of Lung Adenocarcinoma with Long-Term Response after Late-Onset Pembrolizumab-Induced Acute Adrenal Insufficiency. | Secondary adrenal insufficiency | Normal pituitary gland on MRI | Anorexia |
| **Tanabe et al. (2020) (28)** | Differentiating Immune-Related Adrenal Insufficiency From Low Cardiac Output Syndrome: A Case Report. | Secondary adrenal insufficiency | Normal pituitary gland on MRI | Anorexia, nausea, and hypotension |
| **Tanaka et al. (2021) (29)** | Locally advanced endometrial cancer with multiple immune-related adverse events coinciding with the complete response to radiotherapy after immune checkpoint inhibitor therapy: A case report. | Secondary adrenal insufficiency | Empty sella syndrome | Fatigue and anorexia |
| **Yamagata et al. (2020) (30)** | Progression of Hypopituitarism and Hypothyroidism after Treatment with Pembrolizumab in a Patient with Adrenal Metastasis from Non-small-cell Lung Cancer. | Secondary adrenal insufficiency | Normal pituitary gland on MRI | Anorexia, fatigue |
| **Yamamoto et al. (2020) (31)** | Isolated adrenocorticotropin deficiency induced by pembrolizumab for hypopharyngeal cancer | Secondary adrenal insufficiency | Normal pituitary gland on MRI | 3-day-long fever, anorexia, and vomiting |
| **Yong et al. (2023) (32)** | Pembrolizumab-related central diabetes insipidus | Central diabetes insipidus | Neuroimaging weighted images without an infiltrative lesion | Dyspnea, preceded by a 2-week history of malaise |

**References**

1.	Alasmar A, San SS, Ta T, Mani SE. Central Diabetes Insipidus Following Steroid Replacement for Hypoadrenalism in Pembrolizumab-Induced Hypophysitis. Cureus. 2026;18(4):e107752.

2.	Alfares K, Han HJ. Pembrolizumab-Induced Isolated Adrenocorticotropic Hormone (ACTH) Deficiency . Cureus. 2024;16(1):e52235.

3.	Baldawi MB, Dhami B, Gosai J, Al-Khafaji RH. Adverse Endocrine-Related Effects of Pembrolizumab Precipitating Severe Hyponatremia. Cureus. 2022;14(8):e28393.

4.	Balti E, Verhaeghe S, Kruse V, Roels S, Coremans P. Exploring a New Entity of Single-Agent Pembrolizumab-Associated Hypophysitis. Cureus. 2022;14(8):e27763.

5.	Cheng NH, Lee H, Balchander D, Mimms R, Krishnamurthy M. Immunotherapy Induced Adrenal Insufficiency: An Underdiagnosed Cause of Persistent Hypotension in Cancer. J Community Hosp Intern Med Perspect. 2024;14(4):68-70.

6.	Chowdhury RH, Hasan MN. Pembrolizumab Induced Hypophysitis in a Patient Allergic to Triamcinolone: An Increasingly Recognized Endocrinopathy Related to Immunotherapy. Mymensingh Med J. 2020;29(4):1021-5.

7.	Doodnauth AV, Klar M, Mulatu YS, Malik ZR, Patel KH, McFarlane SI. Pembrolizumab-Induced Hypophysitis With Isolated Adrenocorticotropic Hormone (ACTH) Deficiency: A Rare Immune-Mediated Adverse Event. Cureus. 2021;13(6):e15465.

8.	El Alam A, Fleifel M, El Masri D, Nassani BM, Abou Chaaya J, Minkailou M, et al. A Case of Myxedema Coma and Adrenal Insufficiency Post Pembrolizumab. Case Rep Endocrinol. 2024;2024:5444975.

9.	Escano L, Desai P. Secondary Adrenal Insufficiency as an Immune-Related Adverse Event of Pembrolizumab Therapy. Cureus. 2024;16(7):e65163.

10.	Fernando A, Mittal A, Cheema R. Nothing Great Comes Without Its Risks: A Rare Case of Pembrolizumab-Induced Hypophysitis. JCEM Case Rep. 2024;2(1):luad135.

11.	Fujimiya T, Azuma K, Togashi Y, Kuwata K, Unezaki S, Takeuchi H. Pembrolizumab-induced secondary adrenal insufficiency due to adrenocorticotrophic hormone deficiency in a patient with non-small-cell lung carcinoma: a case report. J Pharm Health Care Sci. 2024;10(1):10.

12.	Hashinokuchi A, Haro A, Minagawa R, Haruta Y, Kawano H, Suehiro T, et al. Hypothyroidism With ACTH Deficiency During Pembrolizumab Therapy for Lung Cancer: Case Report and Literature Review. Cancer Diagn Progn. 2023;3(4):498-503.

13.	Hinata Y, Ohara N, Sakurai Y, Koda R, Yoneoka Y, Takada T, et al. Isolated Adrenocorticotropic Hormone Deficiency Associated with Severe Hyperkalemia During Pembrolizumab Therapy in a Patient with Ureteral Cancer and an Ileal Conduit: A Case Report and Literature Review. Am J Case Rep. 2021;22:e931639.

14.	Iijima H, Sakai A, Ebisumoto K, Yamauchi M, Teramura T, Yamazaki A, et al. Metachronous immune-related adverse events involving type 1 diabetes and isolated adrenocorticotropic hormone deficiency associated with pembrolizumab monotherapy for metastatic head and neck cancer: a case report. J Med Case Rep. 2023;17(1):387.

15.	Inoue S, Tsunoda Y, Yamamoto K, Okamoto H. Immunoglobulin G4-related Autoimmune Pancreatitis and Hypopituitarism Following Immune Checkpoint Inhibitor Therapy: A Case Report. Intern Med. 2024.

16.	Iwamoto Y, Tatsumi F, Ohnishi M, Katakura Y, Kimura T, Shimoda M, et al. A case of Empty Sella syndrome with adrenal insufficiency masked by prednisolone after administration of immune checkpoint inhibitors. Medicine (Baltimore). 2024;103(10):e37204.

17.	Kafley S, Tamrakar S, Samra M, Shanmugar S, Gupta I. Immune Checkpoint Inhibitors and Endocrine Disruption: A Case of Hyponatremia and Adrenal Insufficiency. Cureus. 2024;16(9):e70089.

18.	Khan WJ, Asif M, Chaudhry HS, Aslam S, Nadeem I. Pembrolizumab-Induced Broken Heart Syndrome, Pneumonitis, and Hypophysitis Occurring Concurrently; A Deadly Triad. J Community Hosp Intern Med Perspect. 2023;13(3):88-90.

19.	Khatri S, Alom M, Kashfi S, Atallah J, Goswami G. Simultaneous Presentation of Secondary Adrenal Insufficiency and Primary Hypothyroidism due to Pembrolizumab: A Case Report. J Investig Med High Impact Case Rep. 2023;11:23247096231194401.

20.	Montero Perez O, Sanchez Escudero L, Guzman Ramos MI, Avino Tarazona V. Hypophysitis secondary to pembrolizumab: a case report and review of the literature. Anticancer Drugs. 2022;33(1):94-9.

21.	Moreira I, Vilarica AS, Alves P. First report of a very rare complication in a case of nonsmall cell lung carcinoma. Breathe (Sheff). 2021;17(4):210107.

22.	Nakagami C, Endoh M, Nakatsuka M, Nakahashi K, Ota H, Aso M, et al. Pituitary hypoadrenocorticism and hypothyroidism after immunochemotherapy followed by salvage surgery for lung cancer: a case report. Gen Thorac Cardiovasc Surg Cases. 2023;2(1):7.

23.	Nakamura T, Imai R, Nishimura N. A Case of Nonsmall-Cell Lung Cancer with Anaphylaxis after 41 Courses of Pembrolizumab along with Adrenal Insufficiency as an Immune-Related Adverse Event. Case Rep Oncol. 2022;15(3):804-8.

24.	Oguz SH, Unluturk U, Aksoy S, Erbas T. Clinical course and management of pembrolizumab-associated isolated adrenocorticotrophic hormone deficiency: a new case and literature review. Immunotherapy. 2021;13(14):1157-63.

25.	Oristrell G, Baneras J, Ros J, Munoz E. Cardiac tamponade and adrenal insufficiency due to pembrolizumab: a case report. Eur Heart J Case Rep. 2018;2(2):yty038.

26.	Rossi S, Silvetti F, Bordoni M, Ciarloni A, Salvio G, Balercia G. Pembrolizumab-induced Thyroiditis, Hypophysitis and Adrenalitis: A Case of Triple Endocrine Dysfunction. JCEM Case Rep. 2024;2(11):luae200.

27.	Sonehara K, Tateishi K, Hirabayashi T, Araki T, Ikuyama Y, Machida R, et al. A Case of Lung Adenocarcinoma with Long-Term Response after Late-Onset Pembrolizumab-Induced Acute Adrenal Insufficiency. Case Rep Oncol. 2021;14(1):1-7.

28.	Tanabe J, Watanabe N, Ito M, Kanasaki K, Tanabe K. Differentiating Immune-Related Adrenal Insufficiency From Low Cardiac Output Syndrome: A Case Report. Cureus. 2022;14(11):e31349.

29.	Tanaka Y, Amano T, Takahashi A, Nishimura H, Yamanaka H, Yoneoka Y, et al. Locally advanced endometrial cancer with multiple immune-related adverse events coinciding with the complete response to radiotherapy after immune checkpoint inhibitor therapy: A case report. Gynecol Oncol Rep. 2023;49:101265.

30.	Yamagata S, Kageyama K, Takayasu S, Asari Y, Makita K, Terui K, et al. Progression of Hypopituitarism and Hypothyroidism after Treatment with Pembrolizumab in a Patient with Adrenal Metastasis from Non-small-cell Lung Cancer. Intern Med. 2019;58(24):3557-62.

31.	Yamamoto K, Tokumasu K, Oka K, Hasegawa K, Otsuka F. Isolated adrenocorticotropin deficiency induced by pembrolizumab for hypopharyngeal cancer: A case report. Clin Case Rep. 2021;9(6):e04305.

32.	Chen Han Yong AA, Parind Vora, Christopher Hocking. Pembrolizumab related central diabetes insipidus. Journal of Clinical Review & Case Reports. 2023;8(3):64-6.
